# Genetic Analysis of Natural Variation in *Antirrhinum* Scent Profiles Identifies BENZOIC ACID CARBOXYMETHYL TRANSFERASE As the Major *Locus* Controlling Methyl Benzoate Synthesis

**DOI:** 10.3389/fpls.2017.00027

**Published:** 2017-01-19

**Authors:** Victoria Ruiz-Hernández, Benjamin Hermans, Julia Weiss, Marcos Egea-Cortines

**Affiliations:** Genetics, Instituto de Biotecnología Vegetal, Universidad Politécnica de CartagenaCartagena, Spain

**Keywords:** recombinant inbred lines, floral scent, transposable element, *IDLE* MITE, methyl benzoate, acetophenone, β-ocimene, methyl cinnamate

## Abstract

The *Antirrhinum* genus has a considerable complexity in the scent profiles produced by different species. We have analyzed the genetic differences between *A. majus* and *A. linkianum*, two species divergent in the emission of methyl benzoate, methyl cinnamate, acetophenone, and ocimene. The genetic analysis showed that all compounds segregated in a Mendelian fashion attributable to one or two *loci* with simple or epistatic interactions. Several lines lacked methyl benzoate, a major Volatile Organic Compound emitted by *A. majus* but missing in *A. linkianum*. Using a candidate gene approach, we found that the *BENZOIC ACID CARBOXYMETHYL TRANSFERASE* from *A. linkianum* appeared to be a null allele as we could not detect mRNA expression. The coding region did not show significant differences that could explain the loss of expression. The intron-exon boundaries was also conserved indicating that there is no alternative splicing in *A. linkianum* as compared to *A. majus*. However, it showed multiple polymorphisms in the 5′ promoter region including two insertions, one harboring an *IDLE* MITE transposon with additional sequences with high homology to the *PLENA locus* and a second one with somewhat lower homology to the regulatory region of the *VENOSA locus*. It also had a 778 bp deletion as compared to the *A. majus BAMT* promoter region. Our results show that the differences in scent emission between *A. majus* and *A. linkianum* may be traced back to single genes involved in discrete biosynthetic reactions such as benzoic acid methylation. Thus, natural variation of this complex trait maybe the result of combinations of wild type, and loss of function alleles in different genes involved in discrete VOCs biosynthesis. Furthermore, the presence of active transposable elements in the genus may account for rapid evolution and instability, raising the possibility of adaptation to local pollinators.

## Introduction

The study of natural variation in natural populations has a great potential for identifying the genetic structure of complex traits. Studies in plants using natural populations have helped to identify a large number of genes involved in different traits such as flowering time, plant architecture, or biomass production (Alonso-Blanco et al., 2009). One of the major traits in plants is the production of secondary metabolites as it can be considered an interface of interaction with living organisms including bacteria, fungi, other plants, and of course animals. The emission of scent by flowers is considered a key trait to attract pollinators and repel potential pests (Schiestl, 2010).

One of the characteristics of floral scent is the complexity in terms of the number of independent Volatile Organic Compounds (VOCs). Some plants such as roses have over 500 VOCs (Spiller et al., 2010), and there are over 1700 compounds identified in floral scent (Knudsen et al., 2006). The genetic studies on scent and volatiles have been an important part of plant biotechnology as the major compounds involved in flavor and aroma are VOCs. As a result, studies in crops such as tomato, peach, rice or strawberry are well-developed (Zorrilla-Fontanesi et al., 2012; Sánchez et al., 2013; Rambla et al., 2014; Golestan Hashemi et al., 2015). The genetics of scent emission and its control has been studied in a variety of plants with different outcomes. Several components of the complex scent profiles of roses have been resolved to single Mendelian *loci* of one or two genes involved in the synthesis of single VOCs such as nerol, neryl acetate, and geranyl acetate (Spiller et al., 2010). A similar situation has been identified in *Mimulus*. The differences in pollinator choice in bumblebee-pollinated *Mimulus lewisii* and hummingbird-pollinated *M. cardinalis* are the result of changes in three volatiles: D-limonene, β-myrcene, and E-β-ocimene (Byers et al., 2014a). The genetic differences lie in two *loci* coding for a *LIMONENE-MYRCENE SYNTHASE* and an *OCIMENE SYNTHASE* (Byers et al., 2014b). A different situation has been described in Petunia. The Petunia genome has a multi *locus* island involved in the control of scent emission, floral visible color, UV absorption, pistil length, and stamen length (Hermann et al., 2013). Differences between the scented *Petunia axillaris* and the unscented *P. exserta* lie on two *loci*. One is a single *MYB* gene allelic to *ODORANT1* (*ODO1*) involved in the activation of phenylpropanoids synthesis (Klahre et al., 2011), pointing to a possible evolution at the regulatory level. There is emerging evidence from Petunia and *Antirrhinum* that scent and floral size may share some corregulators such as *ENHANCER OF BENZENOID II* in Petunia, involved in scent emission, flower opening, and anthesis (Colquhoun et al., 2011; Van Moerkercke et al., 2011). In *Antirrhinum* the gene *COMPACTA* is involved in maintenance of B function affecting petal size and scent emission (Manchado-Rojo et al., 2012). A study of narrow sense heritability in *Brassica rapa* has shown that although scent profiles between species differ in many cases in a number of independent VOCs, they may have coregulation between them and with other morphological traits or flowering time (Zu et al., 2016). The genes involved in single compound biosynthesis together with regulatory *loci* with several genetic functions indicates that the natural variation of scent emission may identify both regulatory *loci* as in Petunia and structural genes involved in discrete VOCs biosynthesis, or form part of larger pathways, affecting downstream products.

The systematic identification of enzymes responsible for the biosynthesis of many volatiles and secondary metabolites has followed a standard protocol. Coding regions have been expressed in a heterologous system such as bacteria, yeast or plant cells, and the enzyme activity has allowed the identification of the corresponding basic biochemical properties such as Km for different substrates. Such studies have determined enzyme activities involved in biosynthesis of alkaloids, phenylpropanoids, benzenoids etc. (Murfitt et al., 2000; Collu et al., 2001; Dudareva et al., 2003). However, the identification of genetic variation and the corresponding allelic differences allowing unequivocal annotation of the different coding genes in plants is lagging behind. This is especially important as some enzymes have been found to be able to produce more than one product *in vitro* and may act on different substrates *in vivo*.

The genus *Antirrhinum* comprises roughly 28 species with a center of origin in the Iberian Peninsula (Vargas et al., 2009). Work in *Antirrhinum* has shown that methyl benzoate, myrcene, and nerolidol are produced in a circadian fashion (Kolosova et al., 2001a; Dudareva et al., 2005). The complexity of scent components is reflected in the identification of at least 120 VOCs previously described in plants (Weiss et al., 2016b). In this work, we have performed a genetic analysis of scent emission spanning three generations following a cross of *A. majus* and *A. linkianum*. Both species differ in the production of four VOCS: methyl benzoate, β-ocimene, methyl cinnamate, and acetophenone. These compounds displayed Mendelian segregations typical for a single gene or two *loci* in the F2 population. We identified a loss of function allele of *BENZOIC ACID CARBOXYMETHYL TRANSFERASE* (*BAMT*), a gene involved in methyl benzoate synthesis in higher plants. The null allele is the result of a genomic insertion in the promoter region that was likely mediated by an *IDLE* MITE transposable element (Cartolano et al., 2007) in conjunction with additional genomic rearrangements including a second insertion of genomic sequences with similarity to the *VENOSA locus* (Schwinn et al., 2006). The underlying activity of transposable elements may represent a mechanism for the rapid evolution of scent profiles by promoting genomic rearrangements in key VOC biosynthetic enzymes or their regulatory elements.

## Materials and methods

### Plant material and growth conditions

Plants of *Antirrhinum majus* and *A. linkianum* were grown as described previously as a single plant per pot allowing maximum number of flowers to be produced (Weiss et al., 2016a). We performed a cross between *A. majus* line 165E and *A. linkianum* to obtain a recombinant inbred line.

### VOC collection

Plants belonging to the F2 or F3 segregating population were sampled once and in those cases where little or no scent was obtained a second resampling was performed. Flowers were incubated in a 25 ml glass beaker containing 4 ml of 5% glucose. The pedicel was in contact with the solution. The glass beakers were placed inside 1 l glass desiccators under a regime of 12:12 light dark and 23–18°C conditions. Samples were taken for 24 h periods. Flowers were weighted before and after the scent collection. Quantification of scent emission was based on flower total emission.

### Gas-chromatography mass spectrometry

Trapped floral volatiles were analyzed by gas chromatography–mass spectrometry (GC-MS) as described (Manchado-Rojo et al., 2012). Data analysis and volatile identification was performed with the MSD ChemStation (Agilent Technologies) software.

For semi-quantifying the main VOC compounds of the RILs (methyl benzoate, methyl cinnamate, acetophenone, and β-ocimene) we used standard solutions (Sigma-Aldrich products codes: 18344, 96410, 42163, W353901) diluted with methanol. The concentration of β-ocimene ranged from 25 to 1250 ppm, whereas the concentration of the rest of compounds ranged from 50 to 2500 ppm. An injection volume of 0.5 μl was applied directly to a Twister™. The standards were directly injected using a split/splitless injector (Agilent Technologies). Calibration curves were calculated by Chemstation (methyl benzoate: 1.181^*^10^7^x − 1.009^*^10^5^, *r*^2^ = 0.999; methyl cinnamate: 1.762^*^10^7^x − 5.245^*^10^5^, *r*^2^ = 1; acetophenone: 1.052^*^10^7^x − 2.693^*^10^5^, *r*^2^ = 0.999; β-ocimene (Z): 8.318^*^10^6^x − 1.397^*^10^5^, *r*^2^ = 0.999). The corresponding calibration curves were used to quantify the major compounds segregating. Total amounts are given in μg·flower^−1^·24 h^−1^ and in μg·fresh weight (fw) ^−1^·24 h^−1^.

### Cloning of *A. linkianum BAMT*

We obtained sequence information of *Antirrhinum majus* and developed PCR primers to amplify the genomic region corresponding to BAMT (AF198492.1). We amplified the complete coding region and 2.1 kb corresponding to the 5′ region upstream of the coding region from *A. majus 165E* and *A. linkianum* (Table S1) using TAKARA PrimeStar GXL TAQ polymerase. The amplified DNA fragments were T/A cloned in pGEMTEasy according to the manual and grown in DH10B *E. coli*. DNA sequence was determined by Sanger using standard primers for pGEMTeasy. The accession numbers of the sequences are KU512977 *A. majus BAMT* and KU512978 for *A. linkianum BAMT*.

### qPCR

We analyzed the steady state accumulation of transcripts coding for *BAMT* by qPCR as described before (Delgado-Benarroch et al., 2009). Petals were sampled at T6 subjective time being T0 dawn, as it coincides with the highest *AmajusBAMT* expression (Kolosova et al., 2001a). Total RNA was extracted from fully developed petals using the RNAEasy Kit from Macherey and Nagel according to the instructions. Genes were amplified in a Stratagene Mx3000 qPCR machine (www.agilent.com), with sequence-specific primers (Table S1) synthesized by Invitrogen (www.invitrogen.com) using Takara SYBR-Green (www.thermofischer.com). We used the gene *UBIQUITIN CONJUGATING ENZYME2* (accession number AJ560266.1; Bey et al., 2004) as a control for normalization. The PCR program was performed with 45 cycles including a 10 min denaturation at 95° followed by 30 s at 56°, 45 s at 72°, and 30 s at 95°. We performed experiments with one biological replica and two technical replicas for each of the lines analyzed. The experiment was repeated twice including mRNA extractions with similar results.

## Results

### Construction of recombinant inbred lines

**Figure 1:**
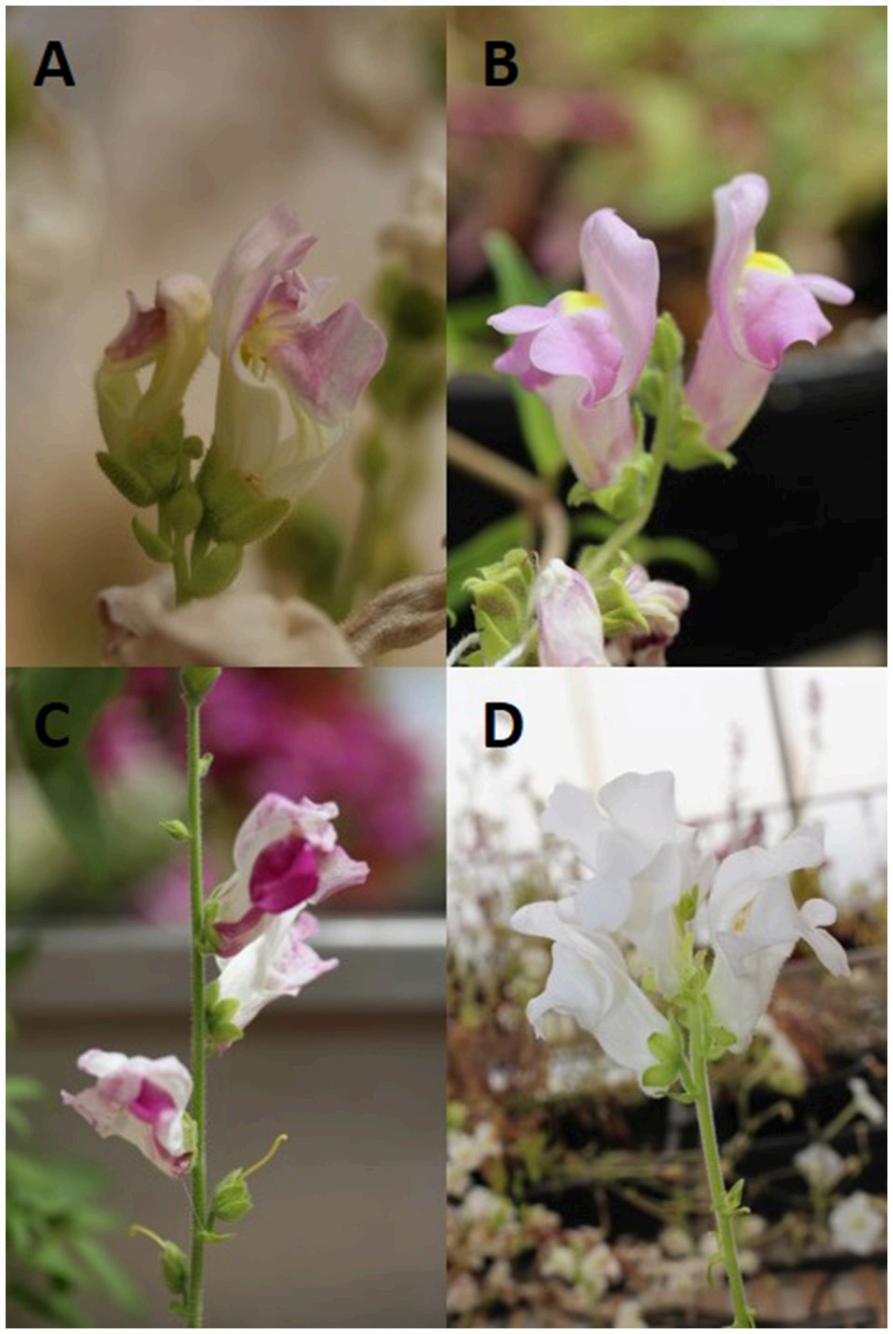
**Pictures of flowers of (A)** *Antirrhinum majus* 165E **(B)** *A. linkianum* and two F2 siblings differing in color. **(C)** The transposon activity could be noted and **(D)** showing white flowers.

We had previously found that *Antirrhinum majus* 165E and *A. linkianum* differ in the emission of four main floral scent VOCs, methyl benzoate, methyl cinnamate, β-ocimene, and acetophenone (Weiss et al., 2016b). In order to identify the genetic components involved in the differential emission, we constructed an F2 from *A. majus* line 165E × *A. linkianum* (Figure 1). The *A. majus* line 165E has a strong activity of transposable elements and is mutated in the *PALLIDA locus* coding dihydroflavonol-4-reductase (Martin et al., 1985). This enzyme catalyzes the reduction of dihydroquercetin to leucocyanidin, during the last steps in anthocyanin biosynthesis thus allowing the visual monitoring of transposon activity in the flowers.

We obtained an initial population of 174 F2 plants. We used a total of 110 F2 plants that flowered during a period of 5 months to analyze scent production and selfed the plants to obtain further inbred lines.

### Genetic analysis of scent profiles

**Figure 2:**
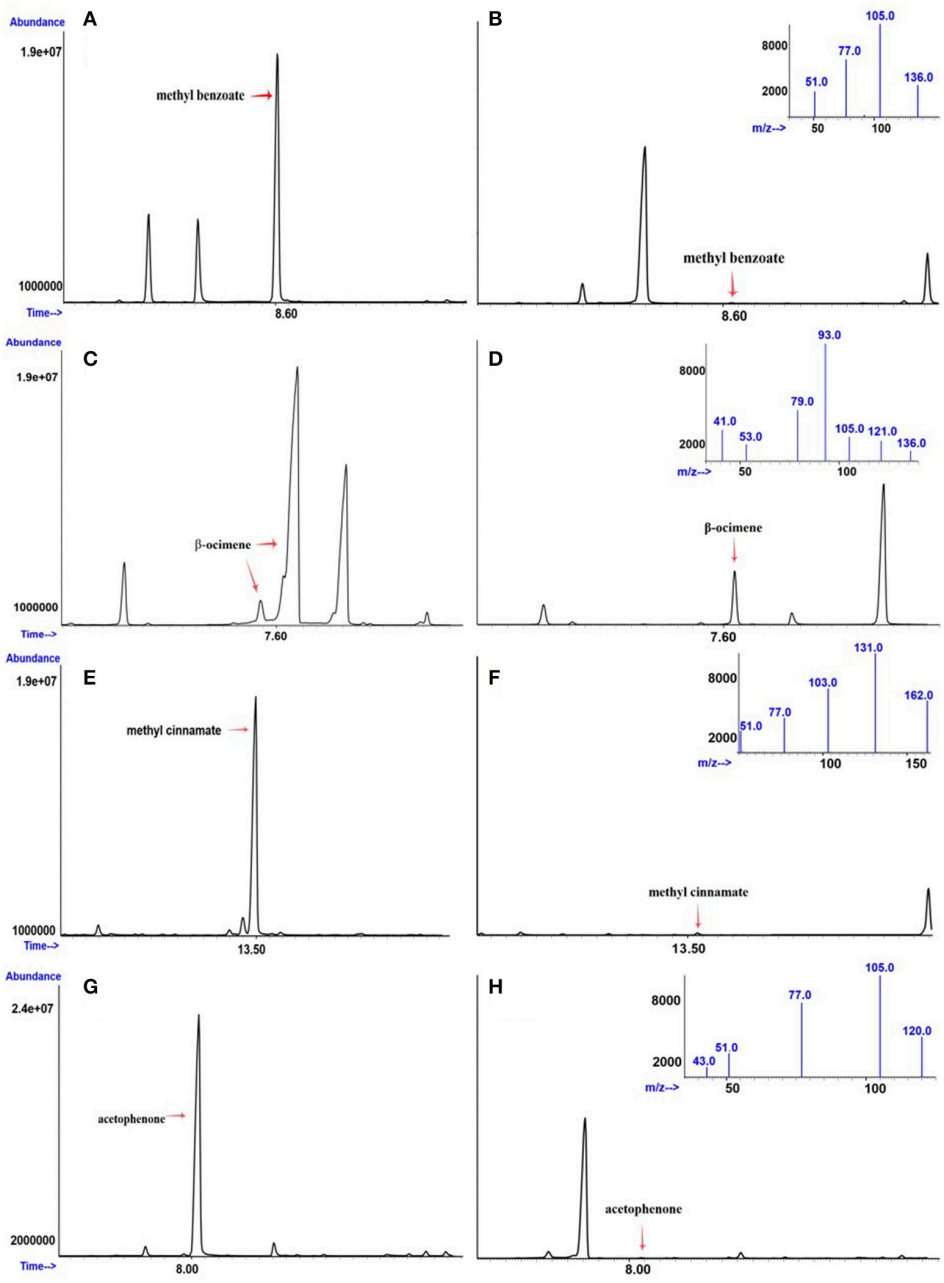
**Gas chromatogram and mass spectra data of plants differing in the emission of (A)** high and **(B)** low methyl benzoate; **(C)** high and **(D)** low β-ocimene; **(E)** high and **(F)** low methyl cinnamate; and **(G)** high and **(H)** low acetophenone. The chromatograms reflect the different signal abundances as peaks. The *X*-axis corresponds to retention times in minutes.

We identified a number of individuals in the F2 that produced either low or close to undetectable levels of the four VOCs that are contrasting between the parental lines (Table 1). We analyzed the segregation of the compounds and found a range of methyl benzoate emission between 203.41 μg·flower^−1^·24 h^−1^ from line 39 and down to 1.12 μg·flower^−1^·24 h^−1^ from line 1 (Figure 2). The emission of β-ocimene ranged between 372.91 μg·flower^−1^·24 h^−1^ from line 4 and 1.85 μg·flower^−1^·24 h^−1^ from line 96. The emission of methyl cinnamate ranged between 48.07 μg·flower^−1^·24 h^−1^ from line 102 and was 0.03 μg·flower^−1^·24 h^−1^ from lines 16, 29, 31, 36, 43, 62, 86, 108, and 112. Finally, acetophenone emission ranged between 304.29 μg·flower^−1^·24 h^−1^ from line 19 and down to 0.03 μg·flower^−1^·24 h^−1^ from line 47.

**Table 1:** ***A. majus* × *A. linkianum* (F2) emission of methyl benzoate, β-ocimene, methyl cinnamate, and acetophenone (μg·FLOWER^−1^·24 h^−1^)**.

**Line**	**Methyl benzoate**	**β-ocimene**	**Methyl cinnamate**	**Acetophenone**
1	1.12	37.35	0.20	0.74
2	42.91	103.67	4.26	10.06
3	3.69	175.77	0.56	186.78
4	53.32	372.91	15.48	260.36
5	37.56	92.09	0.30	32.01
6	15.72	11.64	0.39	2.61
7	13.17	97.24	0.46	91.23
8	6.98	79.04	15.20	2.98
9	1.22	94.81	3.52	0.26
10	126.67	100.64	1.08	11.95
11	123.12	35.91	0.43	75.31
12	30.62	32.59	0.86	9.43
13	40.25	31.57	23.67	303.03
15	80.92	57.42	7.04	66.73
16	1.39	7.99	0.03	0.49
18	54.50	51.17	0.29	27.95
19	17.13	186.93	2.19	304.29
21	5.25	97.26	0.52	0.15
22	44.19	113.57	0.08	11.94
23	47.14	96.41	0.10	88.92
24	37.50	29.06	0.74	29.53
25	11.08	5.62	0.75	40.70
27	0.56	82.47	3.10	0.21
29	14.30	53.98	0.03	48.07
31	43.09	29.12	0.03	14.26
32	1.05	65.38	1.25	0.77
33	11.92	71.76	0.12	35.70
34	1.14	11.89	5.25	0.88
35	38.17	3.28	1.09	74.16
36	2.01	1.95	0.03	2.38
37	94.29	210.09	13.99	3.70
38	2.24	76.58	0.09	1.51
39	203.41	325.85	4.04	12.29
40	42.63	36.71	1.50	88.72
41	15.59	32.86	2.25	19.46
42	67.66	32.76	1.11	24.65
43	66.69	143.26	0.03	0.56
44	37.82	72.15	0.15	160.01
47	146.77	284.77	35.47	0.03
48	74.83	67.91	45.13	25.83
51	162.06	236.10	2.42	246.65
52	11.64	3.89	0.08	7.11
53	58.37	20.86	0.09	53.34
56	3.75	5.25	22.78	1.65
57	43.21	69.50	1.77	19.54
58	60.24	7.48	1.76	79.76
60	26.40	4.73	0.19	20.00
62	3.71	28.65	0.03	25.30
64	2.52	75.33	2.50	0.85
65	4.55	78.02	0.23	14.31
66	74.04	105.48	2.38	125.28
67	2.87	97.26	0.27	4.94
68	54.99	138.41	38.11	69.35
69	21.47	14.26	30.28	2.87
71	12.28	73.29	0.99	19.83
72	16.3	13.58	4.75	96.63
73	1.22	21.24	19.07	2.27
74	5.17	240.55	14.42	1.1
75	67.83	298.36	11.63	122.75
76	87.3	126.57	1.55	19.56
78	1.13	91.79	0.81	93.54
79	86.43	81.75	2.05	64.87
80	46.83	2.55	3.77	55.66
83	41.98	199.34	5.08	3.2
85	43.1	130.71	0.1	133.19
86	52.21	103.41	0.03	42.49
87	77.98	199.87	1.75	289.0
88	97.71	220.32	9.82	7.0
89	53.87	74.45	2.43	9.15
90	16.0	84.57	0.37	21.21
91	39.11	62.58	0.84	15.83
93	33.63	199.62	5.55	3.92
95	26.36	23.56	0.14	11.41
96	12.46	1.85	16.44	2.67
97	68.79	52.54	16.42	172.94
98	5.8	51.46	20.51	3.69
99	139.65	51.17	21.95	166.28
100	60.08	22.76	6.12	17.67
101	19.24	28.21	0.8	1.4
102	64.38	31.43	48.07	6.94
103	89.71	146.16	0.2	7.2
104	13.37	3.32	0.33	1.1
105	19.74	63.48	25.82	3.51
106	28.71	90.44	12.03	1.74
107	1.76	54.66	6.81	3.63
108	42.9	79.91	0.03	266.72
109	168.38	148.08	0.1	373.39
110	64.48	128.9	3.33	18.46
111	95.91	22.79	0.6	35.11
112	88.53	118.99	0.03	75.56
113	61.93	41.29	3.41	108.44
114	2.88	166.18	0.12	1.52
115	66.17	85.87	0.08	71.67
116	1.92	2.97	0.37	2.29
117	195.26	82.87	0.2	211.54
118	36.98	4.44	0.66	33.23
119	25.78	30.85	0.76	44.79
120	84.16	99.84	0.13	61.18
121	29.72	104.68	10.87	126.79
122	69.82	87.67	10.87	123.69
123	21.13	78.98	17.6	0.49
124	60.34	154.03	0.08	184.3
125	64.47	193.11	9.51	154.08
126	56.69	188.98	15.91	198.31
127	28.44	56.75	0.1	1.55
129	2.85	252.88	4.71	249.51
130	49.88	114.28	1.68	29.64
131	50.46	50.27	0.08	4.38
132	9.62	6.39	0.06	6.3
133	155.67	197.02	3.89	114.4

**Figure 3:**
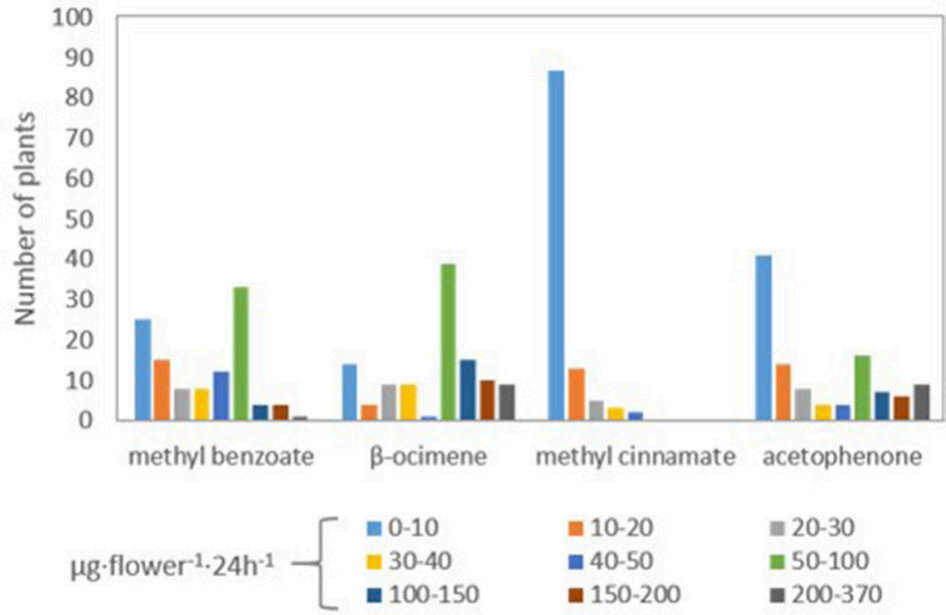
**Quantification of the number of plants emitting different quantities of methyl benzoate, β-ocimene, methyl cinnamate, and acetophenone**.

We analyzed the data of the different scent components to identify a possible genetic model of segregation for each of the VOCS, considering the current knowledge at the biochemical level (Figure 3). The enzyme benzoic acid carboxymethyltransferase (BAMT) is the major enzyme involved in the synthesis of methyl benzoate (Dudareva et al., 2000; Murfitt et al., 2000; Effmert et al., 2005). The segregation analysis of plants producing high and low amounts of methyl benzoate complied to a model of a single gene with Mendelian 3:1 segregation (Chi square test *p* = 0.582), where the plants producing methyl benzoate were dominant (Table 2). This indicated that the allele of *A. linkianum BAMT* could be a major candidate gene involved in the synthesis of methyl benzoate. Furthermore *A. linkianum BAMT* should probably code for a loss of function allele.

**Table 2:** **Statistical analysis of the Mendelian segregation in an F2 population of methyl benzoate, β-ocimene, methyl cinnamate, and acetophenone in a cross of *A. majus* × *A. linkianum* (*n* = 110 plants)**.

**Compound**	**Segregation model**	**Chi square *P*-value**
Methyl benzoate	3:1 (85:25)	0.582
β-ocimene	3:1 (96:14)	0.002953
	3:1 (92:18)	0.03645
	3:1 (83: 27)	0.9123
	13:3 (96:14)	0.1056
Methyl cinnamate	3:1 (87:23)	0.3218
Acetophenone	3:1 (68:41)	0.002354
	1:2:1 (41:46:22)	0.0001789
	9:7 (68:41)	0.1966

The synthesis of β-ocimene in *A. majus* is performed by a specific β-ocimene synthase (Dudareva et al., 2003). The emission of β-ocimene followed a similar pattern as methyl benzoate but in contrast, the number of plants producing low or very low amounts was lower. In fact, we always found small amounts of β-ocimene indicating that a possible allele of *A. majus OCS* was a hypomorphic allele but not a complete null. We tested the hypothesis of a single gene segregating taking into account three different thresholds of emission. The most strict showed significant differences to a 3:1 Mendelian segregation (*p* = 0.002953; Table 2). A slightly less conservative cutoff at levels below 30 μg·flower^−1^·24 h^−1^ did result in acceptable statistical fits (*p* = 0.9.123). Using the strict threshold below 10 μg·flower^−1^·24 h^−1^, we also found a possible model for a 13:3 segregation (*p* = 0.1056) where two genes would come into play (Table 2).

The synthesis of methyl cinnamate is produced by the cinnamate/p-coumarate carboxylmethyltransferases (CCMTs; Kapteyn et al., 2007), in what appears to be a single gene scheme in *Ocium basilicum*. The data inspection showed a clear cut group of 87 plants that produced little or no methyl cinnamate (Figure 2) and a set of 23 that produced substantially higher levels. This data distribution fit perfectly to a 3:1 Mendelian model (*p* = 0.3218; Table 2) albeit with a dominant allele that did not produce methyl cinnamate and a recessive allele that produced this volatile. Thus, there is either a suppressor in *trans* or a dominant negative allele of the corresponding gene in the *A. majus* genetic background.

Finally, the synthesis of acetophenone is thought to be a degradation of ethylbenzene in bacteria. The degradation of 1-phenylethanol is caused by two enzymes a naphthalene dioxygenase and 1-phenylethanol dehydrogenase (Simon et al., 1993; Kniemeyer and Heider, 2001). We found what appeared to be a relative large number of lines producing very little acetophenone (41) indicating a possible effect of two genes. Indeed single gene models were significantly different from a 3:1 (*p* = 0.002354) or 1:2:1 (*p* = 0.0001789; Table 2). However, a 9:7 epistatic segregation was statistically possible (*p* = 0.1966) indicating the probable effect of two genes in the synthesis of acetophenone as proposed in bacteria. As found for β-ocimene, the *A. linkianum* alleles involved in acetophenone synthesis or its control, may not be null alleles resulting in small albeit detectable emissions.

### Coding region of *A. linkianum BAMT*

We decided to analyze the molecular structure of the *A. linkianumBAMT* allele segregating in the population. In order to identify possible lesions responsible for the loss of methyl benzoate emission we followed a candidate gene approach. Previous work has shown that the coding region of benzoic acid carboxymethyl transferase purified from petal tissue of *Antirrhinum* and expressed in *E. coli* can produce methyl benzoate, giving unequivocal biochemical support for the function of the corresponding gene product (Murfitt et al., 2000). Further, analysis of the protein expression has shown that *A. majusBAMT* is expressed in petals (Kolosova et al., 2001b), the tissue that produces methyl benzoate. Despite the biochemical and cellular evidence, there was no genetic evidence that *A. majus BAMT* is the major gene involved in the synthesis of methyl benzoate. This is especially critical as there are two additional enzymes, salicylic acid carboxymethyl transferase (SAMT) and jasmonic acid carboxymethyl transferase (JAMT) with similar structure. Even though SAMT has a low Km for benzoic acid it is able to produce methyl benzoate *in vitro* (Negre et al., 2002; Effmert et al., 2005) and it may be able to produce some methyl benzoate *in vivo*.

**Figure 4:**
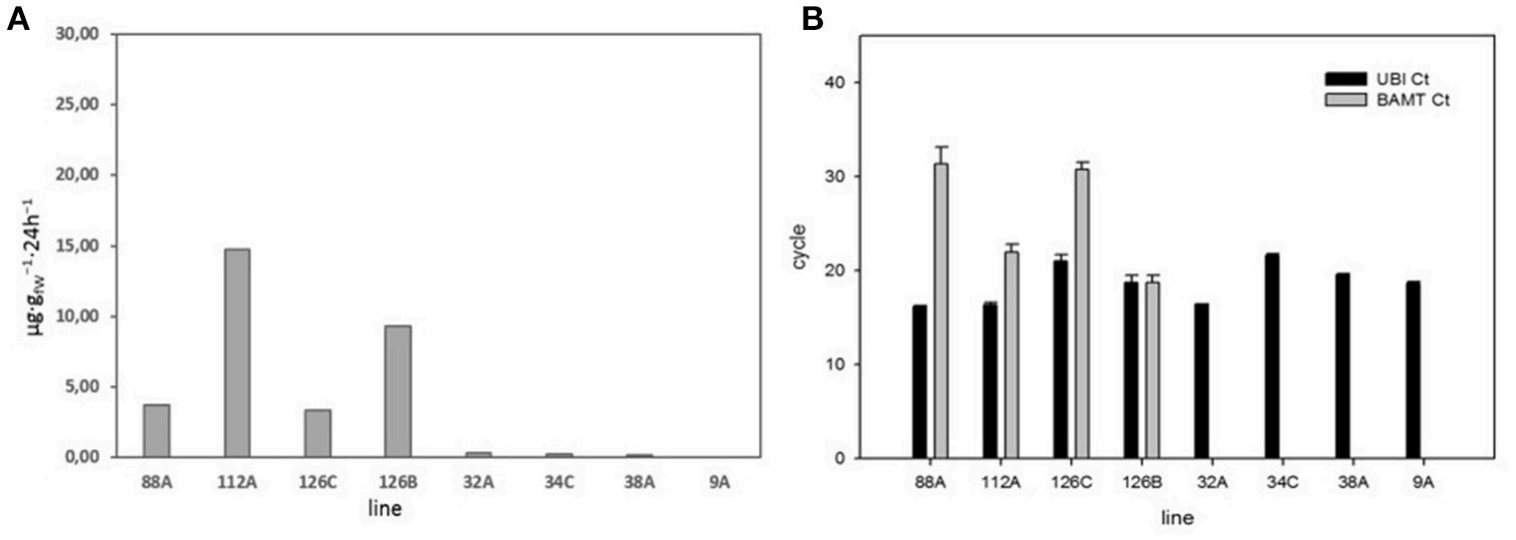
**(A)** Emission of methyl benzoate in eight F3 lines with high and low emissions of methyl benzoate. **(B)** Quantitative PCR of BAMT gene expression of the high and low emitting lines. We used *AmUBC2* as positive control. Results are expressed as where the CT threshold was achieved.

We used the published sequence of *A. majus BAMT* to design primers to amplify the coding region of *A. linkianum BAMT* from gDNA in plants that were not producing methyl benzoate (Table S1). We used petal tissues from F3 plants producing large amounts of methyl benzoate and very low to undetectable levels (Figure 4). We could not detect *A. linkianum BAMT* expression in plants that did not emit methyl benzoate (Figure 4). The PCR primers developed to amplify a fragment of *A. linkianum* and *A. majus* turned out to be in a conserved region of the cDNA corresponding to the fourth exon, suggesting that the lack of amplification was not due to lack of annealing of the designed primers (Supplementary Figure 1).

**Figure 5:**
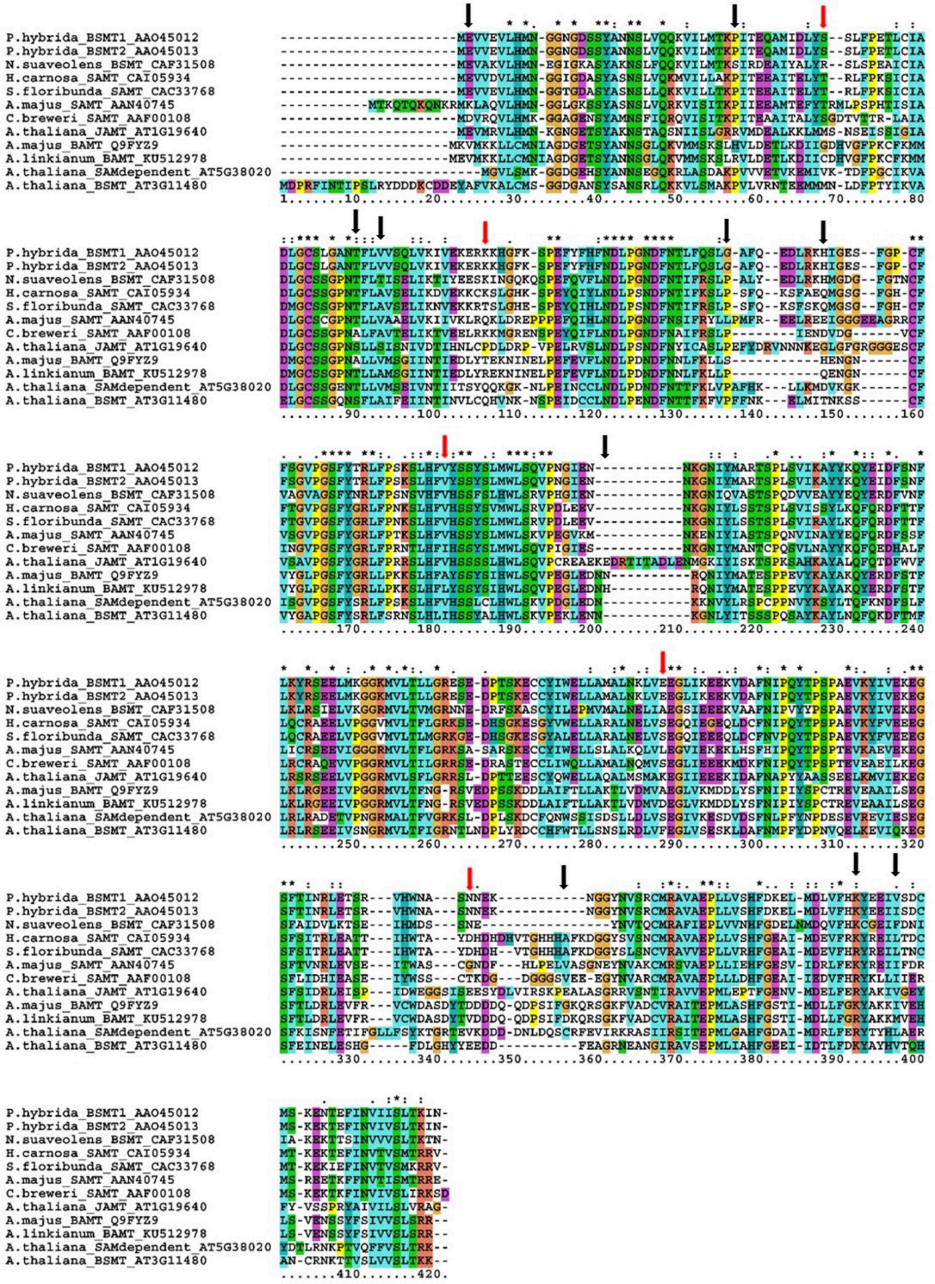
**Multiple sequence alignment of different SABATH family proteins**. *Petunia hybrida*_BSMT1 (AAO45012), *P. hybrida*_ BSMT2 (AAO45013), *Nicotiana suaveolens*_BSMT (CAF31508), *Hoya carnosa*_SAMT (CAI05934), *Stephanotis floribunda*_SAMT (CAC33768), *Antirrhinum majus*_SAMT (AAN40745), *Clarkia breweri*_BAMT (AAF00108), *Arabidopsis thaliana*_JAMT (AT1G19640), *A. majus*_BAMT (Q9FYZ9), *A. linkianum*_BAMT (KU512978), *A. thaliana*_SAMdependent (AT5G38020), *A. thaliana*_BSMT (AT3G11480). Non-conserved amino acids with *A. linkianum* marked with red arrows, conserved amino acids with black arrows. Alignment performed with CLUSTALX (Larkin et al., 2007). Colors are default CLUSTALX color codings (Procter et al., 2010), corresponding to: blue hydrophobic; red positively charged, purple negatively charged, yellow small (P), cyan (Y and H), green polar, and orange (G). ^*^Are conserved positions and: indicate amino acid conservative changes >0.5 in the Gonnet PAM 250 matrix, and a . indicates weak conservation <0.5 in the Gonnet PAM 250 matrix.

The *BAMT* coding sequence in *A. majus* is composed of four exons (1110 bp). As the expression of *BAMT* in plants that do not produce methyl benzoate was undetectable, we used the genomic DNA of the parental *A. linkianum* to sequence the *A. linkianum BAMT* allele. We found a total of 40 SNPs in the coding region as compared to *A. majus*. These polymorphisms at the DNA level caused 15 amino acid changes at the protein level (Figure 5; Supplementary Figure 1). The mutations identified fell into two groups, one of conserved amino acid changes inside the BAMT/SAMT family of proteins and a second one of non-conserved amino acids. There were 10 amino acid changes in the first group but all the mutations corresponded to conservative amino acid substitutions. The amino acid His36 of *A. majus* BAMT is a proline in the proteins analyzed (Figure 5, Table 3) but was substituted by Arg in *A. linkianum* BAMT, that is conserved in the *Arabidopsis thaliana* JAMT (AT1G19640). The Ala69 from *A. majus* BAMT was conserved with *Clarkia breweri* SAMT but was substituted by a Thr in *A. linkianum* BAMT, that is conserved in most of the species analyzed. The second group of mutations comprised five amino acid changes in positions that are not conserved in the SHABAT proteins. The amino acid differences did not explain the complete lack of mRNA expression, indicating a different source of change.

**Table 3:** **Aminoacid polymorphisms between *A. linkianum* and *A. majus* BAMT proteins and their position**.

	**Aminoacid**														
*A. majus*	K	H	**G**	A	V	**T**	S	H	**A**	N	**A**	**D**	G	K	I
*A. linkianum*	E	R	**C**	T	A	**R**	P	Q	**L**	H	**D**	**V**	D	R	M
aa-Position	2	36	**47**	69	72	**85**	115	116	**143**	163	**239**	**292**	303	338	343

*Methionine was in the position 1 in the coding sequence of both proteins. Polymorphisms in bold are non-conserved in BAMT/SAMT proteins*.

We examined the exon-intron boundaries of the *AmajusBAMT* and *AlinkianumBAMT* genes. Both were identical at the exon-intron boundaries, except for a single base pair (596) within the third intron and four base pairs 3′ of the exon-intron boundary predicted by the *AmajusBAMT* annealing of genomic and cDNA sequences (Supplementary Figure 1). Using the *A. linkianum* sequence to predict possible exon-intron boundaries (Huang et al., 2006) for the corresponding interval where the 596 SNP occurs (bp400–700), gave as a result a splicing site identical to the exon-intron structure of *AmajusBAMT*. Thus, an alternative splicing that would produce a cDNA fragment lacking the last two exons, is not probable.

### Genomic structure of *A. linkianum BAMT*

**Figure 6:**
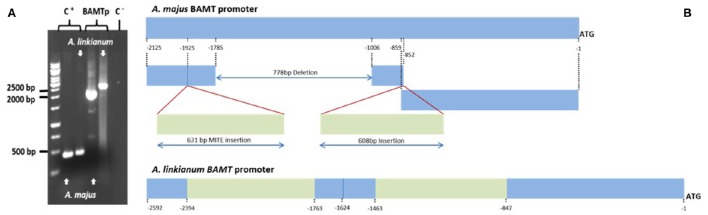
**Comparison of the molecular structure of the *A. majus and A. linkianum BAMT* promoter. (A)** Electrophoresis of PCR product of *BAMT* promoter from *A. majus* and *A. linkianum. PLENA* as a positive control (C+) and water as a negative template (C−). **(B)** *A. majus* and *A. linkianum BAMT* promoters. Fragments in blue show high similarities between *A. majus* and *A. linkianum*. Green fragments show two major insertions in the *A. linkianum BAMT* promoter compared to the *A. majus BAMT* promoter.

We developed additional PCR primers based on the promoter region of *A. majus BAMT* and amplified 2125 bp of the promoter region of *A. majus*, and the corresponding region from the *A. linkianum* genome. The *A. linkianum* promoter was longer (2592 bp; Figure 6A) suggesting major changes in the promoter structure.

**Figure 7:**
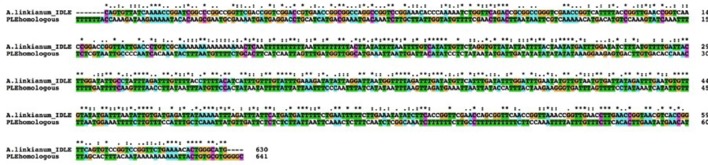
**Alignment of the two regions of the *A. majus PLE* promoter and the *A. linkianum BAMT* promoter showing high homology**. DNA fragments were aligned with CLUSTALX. Coding corresponds to the default CLUSTALX. Color coding corresponds to T green, C magenta, A blue, G orange. ^*^Correspond to conserved bases,: to T–A, C–A, or G–A changes and empty spaces to C–T, G–T, and G–C.

We divided the *A. linkianumBAMT* promoter in six fragments from 5′ to 3′ corresponding to six regions with distinct features (Figure 6B). We numbered the promoter with 1 as the adenine in the ATG of the *BAMT* CDS. The most distal fragment, −2592 to −2394 bp showed high homology to the *A. majus* promoter (−2125 to −1927 bp) and contained 10 SNPs (Supplementary Figure 2). The second fragment of *A. linkianum BAMT* (−2393 to −1763 bp) had a 631 bp insertion comparing to the *A. majus* promoter, located between the −1926 bp and the −1925 bp of *A. majus*. This insertion included genomic DNA that showed an extreme degree of homology (BLASTN e-114) with a fragment found in the promoter region of the *PLENA locus* (Figure 7; Bradley et al., 1993). This insertion contained an *IDLE* MITE transposable element (Figure 6; Cartolano et al., 2007; Schwarz-Sommer et al., 2010). The following fragment (−1762 to −1624 bp) of 138 bp length showed again high homology to the *A. majus* promoter (−1925 to −1786 bp) and had 6 SNPs. There was a 778 bp deletion in the *A. linkianum* promoter comprising the region between −1785 and −1006 bp from the *A. majus* promoter. The following fragment from −1623 to −1463 bp comprised 160 bp homologous to *A. majus* (−1007 to −852 bp) with 6 SNPs and a 4 bp insertion. The following fragment was an insertion of 608 bp comparing to the *A. majus* promoter, immediately after the −852 bp of *A. majus* promoter. This insertion had homology with the *VENOSA* genomic *locus* (Schwinn et al., 2006; BLASTN 2e-19). Furthermore the insertion was flanked by an 8 bp imperfect tandem duplication. Finally, the closest fragment to the start of transcription was −845 to −1 bp, homologous to *A. majus* −851 to −1 bp. It contained 45 SNPs, five deletions of 1–4 bp and three insertions of 1–4 bp.

Altogether the promoter of *A. linkianum BAMT* has two large insertions, one large deletion and several SNPs and indels, indicating that the complexity of changes may be responsible for the complete loss of expression in petals.

## Discussion

The identification of genes involved in the synthesis of scent and VOCs is an important effort that runs in parallel in a variety of plants. In crops, scent related traits include flavor and aroma and have undergone extensive research in many important plants such as rice (Lorieux et al., 1996; Singh et al., 2007; Golestan Hashemi et al., 2015), tomato (Klee, 2010; Klee and Tieman, 2013), strawberry (Zorrilla-Fontanesi et al., 2012), or trees such as peaches (Eduardo et al., 2013). The use of natural variation has helped first to define the genetic structure of the character and second to identify candidate genes involved in scent and volatile emission. Although scents are complex combinations of VOCs, the genetic structure of this trait has turned out to be composed of single genes and both regulatory genes such as the *MYB* gene *ODORANT1* from Petunia (Klahre et al., 2011) and a large number of enzymes have been identified using this approach. In this study we have analyzed the genetic structure of scent emission in two species of *Antirrhinum, A. majus* and *A. linkianum*, differing in the emission of four VOCs, methyl benzoate, methyl cinnamate, acetophenone, and β-ocimene (Weiss et al., 2016b). Our results show that the different VOCs displayed Mendelian segregations imputable to one or two genes.

The results of the segregation analysis coincided with the currently known biochemical models describing the catalytic reactions leading to the formation of the last step in the synthesis of methyl benzoate, β-ocimene, methyl cinnamate, and acetophenone. However, there are several aspects that may obscure the segregation analysis of scent VOCs. First, natural alleles may result in complete loss of function such as the *A. linkianum BAMT* identified in the current work. But small albeit detectable emissions of a compound such as methyl benzoate may be synthesized by a second enzyme such as *A. majus* SAMT, showing low affinity for benzoic acid (Effmert et al., 2005). The capacity to transform benzoic acid by SAMT seems to be enzyme specific as the tomato SAMT SlSAMT has a very low affinity for benzoic acid while the JAMT enzyme involved in the synthesis of methyl jasmonate can readily produce methyl benzoate (Tieman et al., 2010). Thus, the occasional emission of methyl benzoate could be the result of a family of proteins that have major affinities for their major substrate but maybe able to process additional metabolites with similar structures.

A second aspect is that incomplete loss of function i.e., leaky or weak alleles may result in segregations difficult to interpret as setting an emission threshold may not be straight forward. Indeed there are two possible gene models for β-ocimene synthesis, one based on a single gene, that would agree with the current biochemical model based on a single gene (Dudareva et al., 2003), while the second maybe based on two genes. As the *Antirrhinum* genome is not sequenced we cannot conclude if the first model is the correct one and further genetic and molecular analysis is required to resolve this issue.

The emission of methyl cinnamate appears to be recessive and the dominant allele may be a loss of function. The currently proposed biochemical model of methyl cinnamate synthesis is based on enzymes related to SHABAT carboxymethyl tranferases. This indicates that the loss of function maybe the result of a dominant negative translated gene product or the result of a local transposition event causing a double stranded RNA based gene silencing in trans.

The current model of acetophenone biosynthesis is based on the bacterial degradation of ethylbenzene by anaerobic catabolism where the last step is the degradation of 1-phenylethanol (Kniemeyer and Heider, 2001). This model may be conserved in higher plants where isotope labeling has shown that 1-phenylethanol is the major substrate for acetophenone synthesis in *Camellia sinensis* (Dong et al., 2012). This suggests a conservation of the biochemical pathway in higher plants that probably perform this reaction under aerobic conditions. The segregation of acetophenone in the cross of *A. majus* × *A. linkianum* was most likely due to a two gene model, supporting the current evidence in bacteria where two enzymes are required to perform the synthesis of this volatile.

The lack of *BAMT* mRNA in plants that did not emit methyl benzoate led us to use the *A. majus BAMT* sequence to obtain information of the *A. linkianum BAMT locus*. The total of fifteen amino acid changes between *A. majus* and *A. linkianum BAMT* coding sequences is on one hand high, but is also restricted to either conservative changes or changes in non-conserved amino acids within a set of SABATH proteins. The intron-exon boundaries and predicted splicing sites of *A. majus* and *A. linkianum BAMT* were conserved. This indicates that the lack of mRNA of *A. linkianum BAMT* was not caused by alternative splicing differing between both alleles. The *A. majusBAMT* gene expression is circadian regulated (Kolosova et al., 2001a). We had sampled the flowers for mRNA at the highest level of recorded expression, i.e., roughly at T6 of subjective time. As we neither found *A. linkianum BAMT* expression at this point, nor methyl benzoate emission in a 24 h interval, our assumption is that *A. linkianumBAMT* does not produce mRNA at other times of the day.

However, as the mRNA was undetectable in *A. linkianum* or the corresponding siblings we pursued further to analyze the regulatory region of *A. linkianum BAMT*. The mutations found in the promoter of the *A. linkianum BAMT* gene comprise three major changes including what appears to be an event of non-homologous recombination causing an insertion of a 630 bp fragment that is with high probability originated at the *PLENA locus*. The insertion contains an *IDLE* transposable element that is also present in the *PLENA locus* (Cartolano et al., 2007). The second modification is caused by a 608 bp insertion with low homology to the *VENOSA locus* (Schwinn et al., 2006), flanked by two 8 bp imperfect repeats. These type of mutations maybe caused in an original promoter by a patch-mediated double-strand break induction and repair mechanism (Vaughn and Bennetzen, 2014). We cannot determine if the 778 bp deletion occurred linked to the aforementioned events or happened independently. However, small changes such as 4 bp deletions can give raise to weak hypomorphic alleles such as *deficiens chlorantha* (Schwarz-Sommer et al., 1992). So our assumption is that the complex rearrangement of the *A. linkianum* regulatory region creates what appears to be a null allele in terms of mRNA expression. The low emission of methyl benzoate by some of the F2 recessive lines can be explained by the non-specific production of this compound by SAMT (Effmert et al., 2005). Methyl salicylate synthesis is activated in response to stress. The gene encoding for SAMT is induced by salicylic acid and jasmonic acid in *Antirrhinum* petals (Negre et al., 2002). Although the reported Km of SAMT is over 100-fold lower for benzoic acid than salicylic acid it may be responsible for the small amounts of methyl benzoate emission that we could detect in some samples. We have not tested the hypothesis of a direct involvement of *A. majus BAMT* on the synthesis of methyl salicylate or methyl jasmonate, as we have not performed experiments under stress conditions aimed to activate these pathways. Nevertheless, methyl salicylate is amongst the most common floral scent VOCs (Knudsen et al., 2006), Our data shows that complex scent profiles can be resolved to combinations of Mendelian genes involved in synthesis or control of scent components. The high transposon activity of the *Antirrhinum* genus may be involved in the diversity of profiles and may play a role in local adaptation to pollinators.

## Author contributions

VR-H, BH, JW, and ME-C designed experiments; performed experiments; analyzed the data; corrected and approved the final manuscript. VR-H, JW, and ME-C wrote the manuscript.

## Funding

This work was supported by the Ministerio de Ciencia e Innovación-Fondo de Desarrollo Regional (BFU2013-45148-R) to ME-C and JW; and by the Ministerio de Educación Cultura y Deporte (FPU13/03606) to VR-H.

### Conflict of interest statement

The authors declare that the research was conducted in the absence of any commercial or financial relationships that could be construed as a potential conflict of interest.
