# Supplementary material for: Genetic Analysis of Natural Variation in Antirrhinum Scent Profiles Identifies BENZOIC ACID CARBOXYMETHYL TRANSFERASE As the Major Locus Controlling Methyl Benzoate Synthesis
Source: Front Plant Sci. 2017 Jan 19;8:27. doi: 10.3389/fpls.2017.00027 (PMC5244254; doi:10.3389/fpls.2017.00027)
Supplement: Supplementary file 1 [file DataSheet1.pdf]

Supplementary Material

Genetic analysis of natural variation in Antirrhinum scent profiles identifies BENZOIC ACID CARBOXYMETHYL TRANSFERASE as the locus controlling methyl benzoate synthesis

Victoria Ruiz-Hernández, Benjamin Hermans, Julia Weiss, Marcos Egea-Cortines*

*** Correspondence:** marcos.egea@upct.es

1 Supplementary Tables

Table S1. List of primers used. BAMTprom Forward and Reverse were used for amplifying the BAMT promoter in *A. majus* and *A. linkianum* from gDNA. BAMT_F1786 and BAMT_R2408 were used for amplifying a transcribed region of BAMT. PLENA and UBIQUITIN primers were used as positive controls.

Primer Name	Sequence
BAMTprom Forward	AACCGATGAATTTACGCACA
BAMTprom Reverse	TTCTACTCGGGTATAACTTCTAACG
PLENA Forward	AAATGCTTTCCCAGTTGCTCAACTGC
PLENA Reverse	TATCAGGAGCTTGAGCTGCACAATGC
BAMT_F1786	GCTTGTCAAGATGGACGATTTGTAC
BAMT_R2408	TTCCATTACTTCTGCTATAAAAATAGTACT
UBIQUITIN Forward	TGGAGGATGGAAGGACTTTGG
UBIQUITIN Reverse	CAGGACGACAACAAGCAACAG

2 Supplementary Figures

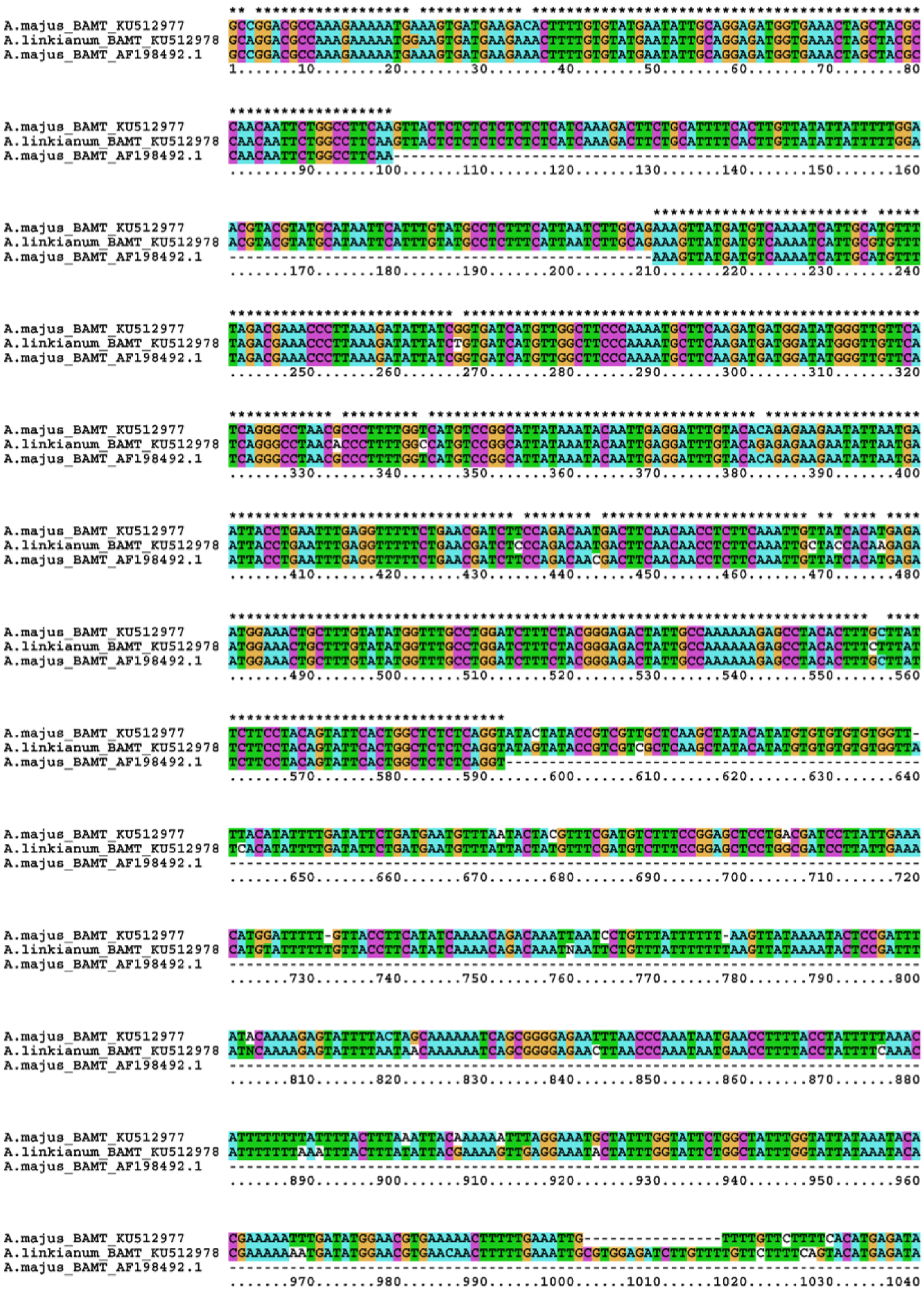

A.majus_BAMT_KU512977
A.linkianum_BAMT_KU512978
A.majus_BAMT_AF198492.1

```
A.majus_BAMT_KU512977       ATTCACTGATTTAATATAACACATGAGGTTACTATGCAACTAGCTCTTAAAGGTATTTTGGGTTCATAGTTTCACATAAA
A.linkianum_BAMT_KU512978   ATTCACTGATTTAATATAACACATGAGGTTACTATGCAACTAGCTCTTAAAGCTATTTTGGGTTCATAGTTTCACATAAA
A.majus_BAMT_AF198492.1     --------------------------------------------------------------------------------
                            ......1050......1060......1070......1080......1090......1100......1110......1120

A.majus_BAMT_KU512977       GAACAAACATATAAAACAAGGCAATATTGAATCAATCATGTATACACACACGCTCACAAACATATAATAATAATAGGAAT
A.linkianum_BAMT_KU512978   GAACAAACATATAAAACAAGGCAATATTGAATCAATCATGTATACACACACGCTCACAAACATATAATAATAATAGGAAT
A.majus_BAMT_AF198492.1     --------------------------------------------------------------------------------
                            ......1130......1140......1150......1160......1170......1180......1190......1200

                                              ********************** ************************* *************
A.majus_BAMT_KU512977       GGGTATTGAATGATAGGTTCCTGAAGGGCTGGAGGATAATAACAGACAAAACATTTACATGGCAACAGAAAGTCCTCCGG
A.linkianum_BAMT_KU512978   GGGTATTGAATGATAGGTTCCTGAAGGGCTGGAGGATAATCACAGACAAAACATTTACATGGCAACGGAAAGTCCTCCGG
A.majus_BAMT_AF198492.1     ------------------TCCTGAAGGGCTGGAGGATAATAACAGACAAAACATTTACATGGCAACGGAAAGTCCTCCGG
                            ......1210......1220......1230......1240......1250......1260......1270......1280

                            **** ***************************************************************************
A.majus_BAMT_KU512977       AAGTATACAAAGCATACGCAAAGCAATACGAAAGAGACTTCTCCACATTTCTAAAGTTGCGAGGCGAGGAAATTGTACCA
A.linkianum_BAMT_KU512978   AAGTATACAAAGCATACGCAAAGCAATACGAAAGAGACTTCTCCACATTTCTAAAGTTGCGAGGCGAGGAAATTGTACCA
A.majus_BAMT_AF198492.1     AAGTGTACAAAGCATACGCAAAGCAATACGAAAGAGACTTCTCCACATTTCTAAAGTTGCGAGGCGAGGAAATTGTACCA
                            ......1290......1300......1310......1320......1330......1340......1350......1360

                            *********** ********************************************************************
A.majus_BAMT_KU512977       GGTGGACGCATTGTCTTGACATTTAACGGCAGAAGTGTTGAAGATCCCTCGAGCAAAGATGACTTAGCAATTTTCACATT
A.linkianum_BAMT_KU512978   GGTGGACGCATGGTCTTGACATTTAACGGCAGAAGTGTTGAAGATCCCTCGAGCAAAGATGACTTAGCAATTTTCACATT
A.majus_BAMT_AF198492.1     GGTGGACGCATGGTCTTGACATTTAACGGCAGAAGTGTTGAAGATCCCTCGAGCAAAGATGACTTAGCAATTTTCACATT
                            ......1370......1380......1390......1400......1410......1420......1430......1440

                            *************** ************* *****
A.majus_BAMT_KU512977       GCTTGCAAAAACACTAGTTGATATGGTGGCTGAGGTATGTACGTGAAATTGTGAATATACAAAGGCTTAGTGATGTCGTC
A.linkianum_BAMT_KU512978   GCTTGCAAAAACACTGGTTGATATGGTGGATGAGGTATGTACGTGAAATTAAGAATATACAAAGGCTTAATGATGTCGTC
A.majus_BAMT_AF198492.1     GCTTGCAAAAACACTAGTTGATATGGTGGCTGAGG---------------------------------------------
                            ......1450......1460......1470......1480......1490......1500......1510......1520

A.majus_BAMT_KU512977       TCGTATTCTAACCATCAATTAATTTATAAGGCCATTTTGCCAAAGGAATAAAAACTTTTTTTGGTGGGAGTGACATAAAT
A.linkianum_BAMT_KU512978   TCGTATTCTAACCATCAATTAATTTATAAGGCCATTTTGCAAAAGGAATAAAAACTTTTTTTGGTGGGAGTGACATAAAT
A.majus_BAMT_AF198492.1     --------------------------------------------------------------------------------
                            ......1530......1540......1550......1560......1570......1580......1590......1600

A.majus_BAMT_KU512977       TGAATAAAACGTGTGAGTCATGAGATCTAGACTAGTTGTGCAATTAAACAATCTTTCAACTATATTTATTGATCTTCATG
A.linkianum_BAMT_KU512978   TGACTAAAACGTGTGAGTCATGAGATCTAGACTAGTTGTGCAATTAAACAATCTTTCAACTATATTTATTGATCTTCATG
A.majus_BAMT_AF198492.1     --------------------------------------------------------------------------------
                            ......1610......1620......1630......1640......1650......1660......1670......1680

A.majus_BAMT_KU512977       TTGTTATTAGGATTTTTAACAGAAATCACGTTTTTTCTTATGTTTTTCTCTTTATTTAGCCCTCAACAAATTAAGGTATA
A.linkianum_BAMT_KU512978   TTGTTATTAGGATTTTTAACAGAAATTAGGTTTTTTCTTATGTTTTTCTCTTTATTGAGCCCTCAACAAATTAAGGTATA
A.majus_BAMT_AF198492.1     --------------------------------------------------------------------------------
                            ......1690......1700......1710......1720......1730......1740......1750......1760
                                                                F1786
                                                    ********************************************************
A.majus_BAMT_KU512977       CAAGAATGAACATTTTGTCTCAGGGGCTTGTCAAGATGGACGATTTGTACTCGTTTAACATTCCTATTTACTCACCATGT
A.linkianum_BAMT_KU512978   CAAGAATGAACATTCTGTCTCAGGGGCTTGTCAAGATGGACGATTTGTACTCGTTTAACATTCCTATTTACTCACCATGT
A.majus_BAMT_AF198492.1     ------------------------GGCTTGTCAAGATGGACGATTTGTACTCGTTTAACATTCCTATTTACTCACCATGT
                            ......1770......1780......1790......1800......1810......1820......1830......1840

                            ********************************************************************************
A.majus_BAMT_KU512977       ACGCGCGAAGTAGAGGCAGCAATTCTGAGTGAAGGGTCTTTTACGTTGGACAGGCTAGAGGTCTTTCGTGTTTGTTGGGA
A.linkianum_BAMT_KU512978   ACGCGCGAAGTAGAGGCAGCAATTCTGAGTGAAGGGTCTTTTACGTTGGACAGGCTAGAGGTCTTTCGTGTTTGTTGGGA
A.majus_BAMT_AF198492.1     ACGCGCGAAGTAGAGGCAGCAATTCTGAGTGAAGGGTCTTTTACGTTGGACAGGCTAGAGGTCTTTCGTGTTTGTTGGGA
                            ......1850......1860......1870......1880......1890......1900......1910......1920

                            ***************** ************ ******************  *****************************
A.majus_BAMT_KU512977       TGCAAGTGACTACACAGATGACGATGATCAGCAAGACCCATCAATCTTTAGCAAACAAAGGAGTGGAAAATTTGTGGCAG
A.linkianum_BAMT_KU512978   TGCAAGTGACTACACAGTTGACGATGATCAACAAGACCCATCAATCTTTGACAAACAAAGGAGTGGAAAATTTGTGGCAG
A.majus_BAMT_AF198492.1     TGCAAGTGACTACACAGATGACGATGATCAGCAAGACCCATCAATCTTTGGCAAACAAAGGAGTGGAAAATTTGTGGCAG
                            ......1930......1940......1950......1960......1970......1980......1990......2000

                            *************************************************************************** ****
A.majus_BAMT_KU512977       ATTGTGTACGGGCTATTACGGAACCAATGCTGGCTAGCCATTTTGGGAGCACTATTATGGATCTTCTATTTGGAAAGTAT
A.linkianum_BAMT_KU512978   ATTGTGTACGGGCTATTACGGAACCAATGCTGGCTAGCCATTTTGGGAGCACTATTATGGATCTTCTATTTGGAAGGTAT
A.majus_BAMT_AF198492.1     ATTGTGTACGGGCTATTACGGAACCAATGCTGGCTAGCCATTTTGGGAGCACTATTATGGATCTTCTATTTGGAAAGTAT
                            ......2010......2020......2030......2040......2050......2060......2070......2080
```

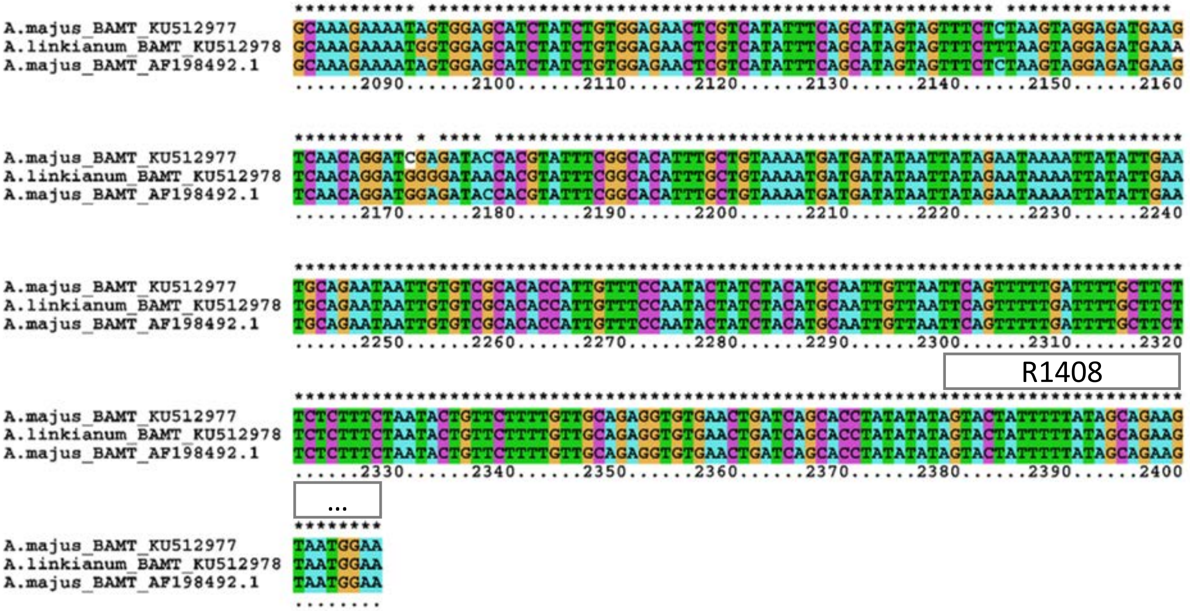

A.majus_BAMT_KU512977
A.linkianum_BAMT_KU512978
A.majus_BAMT_AF198492.1
R1408
...
2090 2100 2110 2120 2130 2140 2150 2160
2170 2180 2190 2200 2210 2220 2230 2240
2250 2260 2270 2280 2290 2300 2310 2320
2330 2340 2350 2360 2370 2380 2390 2400

Supplementary Figure 1. Clustal-X alignment between *A. majus* genomic sequence (KU512977), *A. majus* coding sequence (AF198492.1) and *A. linkianum* genomic sequence (KU512978) of *BAMT*.

```
>A.majus_BAMTp          AACCGATGAATTTACGCACATTTAATAATTCTAAAAACAATTTAAATTTT
>A.linkianum_BAMTp      AACCGATGAATTTACGCACATTTAATAGTTATAAAAAAAAATTTAAAATTT
                        *************************** ** ****** ******** ***

>A.majus_BAMTp          TATTTGGTTAACCATCAATCATCACATGCATTATGATCCTATCATAAATT
>A.linkianum_BAMTp      TATTTCTTTAACCATCAATCATCACACCCATTATGATCCTACCATAAATT
                        *****  *******************  ************* ********

>A.majus_BAMTp          ATTATATATAAATCTAACAATACTATAGAAAGTGACATGTGATTTGGCAC
>A.linkianum_BAMTp      ATTATATATAAATCTAACAATACTATAGAAAGTGACATGTGATTTGGCAC
                        **************************************************

>A.majus_BAMTp          ATAACTAATTATTGACCTTCTGAATGGTCTTGTCCACCCGTGGAGGCATG
>A.linkianum_BAMTp      ATAACTAATTATTGACCTTCTGAATGGTCTTGTCCACCGGTGGAGGCAT-
                        ************************************** **********

>A.majus_BAMTp          --------------------------------------------------
>A.linkianum_BAMTp      CAGTGTTATCAAAACCGGATCGGCCCGGCCGGTTCGACCGGTCGGACCGT

>A.majus_BAMTp          --------------------------------------------------
>A.linkianum_BAMTp      GAACCGGCGGCCAGGCCGGTCCGGAAACACCCAAAAATCTGGTTCAGACC

>A.majus_BAMTp          --------------------------------------------------
>A.linkianum_BAMTp      GCGGCCGGGTCGAACCGGTCATTTTACCGGTTGAACCGGTCAACCGGACC

>A.majus_BAMTp          --------------------------------------------------
>A.linkianum_BAMTp      GGTTATTGACCCTGTCGCAAAAAAAAAAAAAAAACTCAATTTTTTTTTTA

>A.majus_BAMTp          --------------------------------------------------
>A.linkianum_BAMTp      ATTTTTTTTTACTTATATTTTAATTTTGTCATATTGTTCTAGGTGTTATA

>A.majus_BAMTp          --------------------------------------------------
>A.linkianum_BAMTp      TTATTTTACTAATATGATTTGGATATCTTTATGTTTTGATTACTTGGATA

>A.majus_BAMTp          --------------------------------------------------
>A.linkianum_BAMTp      TTGCCTATTTAGATTTGTTTTACCTTTTACATCATTTGTTTGTATTTGAA

>A.majus_BAMTp          --------------------------------------------------
>A.linkianum_BAMTp      AGATATATTAGGATTAATGGTTTTAGATTTGATATGTTCATTTGATATTT

>A.majus_BAMTp          --------------------------------------------------
>A.linkianum_BAMTp      GGATTTGAATATGTTGTAATGTGATTATAGATTTGAATGTGTTGTATATG

>A.majus_BAMTp          --------------------------------------------------
>A.linkianum_BAMTp      ATTTAATATTGTGATGAGATTATAAAAATTTAGATTTATTCATGATGATT

>A.majus_BAMTp          --------------------------------------------------
>A.linkianum_BAMTp      TTTCTGAATTTTTCTTGAAATATATCTTCACCGGTTCGAACCAGCGGTTC

>A.majus_BAMTp          --------------------------------------------------
```

```
>A.linkianum_BAMTp      AACCGGTTAAACCGGTTGAACCTTGAACCGGTAACGTCACCGGTTCAGTG

>A.majus_BAMTp          ------------------------------CATTGCTTAGAAATTTAGAG
>A.linkianum_BAMTp      TCCGGTCCGGTTCTGAAAACACTGGGCATGCATTGCATAGAAATTTAGAG
                                                      ****** *************

>A.majus_BAMTp          CCCCTCAAATTCCTAAAATGCCTTATTAAAGGGGAGGTGGGTTATTGATA
>A.linkianum_BAMTp      CCCCTCAAATTCCTAAAATGCCTTATTAAATAGAAGGTGGGTTATTGATA
                        ******************************  * ****************

>A.majus_BAMTp          TATGTCATGTACAGACCGGAAAGATACCATATAGAAATTTTGAGTCTCCT
>A.linkianum_BAMTp      TATGTCATGTACAGACCGGAATGATACCATATAAAAATTTTGAGTCTCCT
                        ********************* *********** ****************

>A.majus_BAMTp          CAACTCCTAAAAGACCTTGTTGTTCCCTTGGTTGTTCCCTTGTTCAAAAA
>A.linkianum_BAMTp      CAACTCCTAAAAGACCTTAT------------------------------
                        ****************** *

>A.majus_BAMTp          CCACAATAATAAACTAGAGAAATAAAATGATTTTACAACACTTTAACATA
>A.linkianum_BAMTp      --------------------------------------------------

>A.majus_BAMTp          AAATAGATATTACATCAACTCATGAATTGGATGAATGTTACCTCGAAGTC
>A.linkianum_BAMTp      --------------------------------------------------

>A.majus_BAMTp          GATAATTAGTTAATTACCAAAAATGACACAATATAATAGTCAGGATGAGG
>A.linkianum_BAMTp      --------------------------------------------------

>A.majus_BAMTp          GGTGGACTAGGTCTCTGCTCTCTTAAAGACGGTTTTGCGCCCCTACAGAT
>A.linkianum_BAMTp      --------------------------------------------------

>A.majus_BAMTp          TTTTATCCGATGCAGTAGGCTACTGACACGTCCGTCTCCGAGATACAATA
>A.linkianum_BAMTp      --------------------------------------------------

>A.majus_BAMTp          GCCCAACTTTTCTTGTTGTAAACTTCGCTCTAAGTCTGCACTACTTAAGG
>A.linkianum_BAMTp      --------------------------------------------------

>A.majus_BAMTp          AAGTCGAAGCGTAGCCCAACACTTTTCTTTGTCCATAAATCTTTTCTCAT
>A.linkianum_BAMTp      --------------------------------------------------

>A.majus_BAMTp          ATTACACATAAATATAATACTTTTACATATAATAGGTGAAGAACACTTGT
>A.linkianum_BAMTp      --------------------------------------------------

>A.majus_BAMTp          TGAAAGGAGAGTTATATATATATATATATATTTTTTTATGTCTTTTCTCTTA
>A.linkianum_BAMTp      --------------------------------------------------

>A.majus_BAMTp          CTTCAAAATGATATGCAAGACCACTATTTATAGTGTAAAACTCACTAGTC
>A.linkianum_BAMTp      --------------------------------------------------

>A.majus_BAMTp          TTGTAACTCCAAAATAATTAGAGAATTAATATATAATATTATTACACTAT
>A.linkianum_BAMTp      --------------------------------------------------
```

```
>A.majus_BAMTp          ATAAATATGATAGTAATGGGGGTGTTTTAATTCACACAATAAAAGAGGTG
>A.linkianum_BAMTp      --------------------------------------------------

>A.majus_BAMTp          GTAAATGTGAAAGTTAATGGTCTCACAACATTACTCTCATTAAGACCAAT
>A.linkianum_BAMTp      --------------------------------------------------

>A.majus_BAMTp          ACTTGTAACATACAAAATATTTACCCCTACTAACACAAGACAATATAGTC
>A.linkianum_BAMTp      --------------------------------------------------

>A.majus_BAMTp          TTGTAAATTAATCTCAAAATAATTATTTTTATTAAATACGGACCTTACTA
>A.linkianum_BAMTp      ------------------------------------------------TA
                                                                        **

>A.majus_BAMTp          AGGAGAAAATAGAATCTTATTTAATATACCAAACACAAATTCATTCTCAA
>A.linkianum_BAMTp      AGAAGAAAATAGAATGTTAATTAATATACCAAACCCAAATTCCTTCTCAA
                        ** ************ *** ************** ******* *******

>A.majus_BAMTp          GCATTAAT----ATTGCTAGATTTCATCACACAACATTAATTTTAGTTGT
>A.linkianum_BAMTp      GCATTAATTAATATTGCTAGATTTCATCAAACAACATTAATTTTAGTTGT
                        ********    **************** *********************

>A.majus_BAMTp          CCCCTATTTTTCCATTTTCTAAAGGTAGAGTGCTTTCACGTGCGACATGA
>A.linkianum_BAMTp      CCCCTATTTTTCCATTTTCTAAAGGTAGAGTGCTTTCACGTGCGACATGA
                        **************************************************

>A.majus_BAMTp          TTTACACC------------------------------------------
>A.linkianum_BAMTp      TTTTCACCATTAAAAGAATAAAATTGTAAGTAGATTTGTGATGAATTACA
                        *** ****

>A.majus_BAMTp          --------------------------------------------------
>A.linkianum_BAMTp      TCTACTTAACTATATTGAATATTTTGTGATATGCGATACTTTATAAGTAC

>A.majus_BAMTp          --------------------------------------------------
>A.linkianum_BAMTp      TTTTTTTGACTGAAATGGAGGCCGAGAAAATGCCCGGGGCCTCAGATACT

>A.majus_BAMTp          --------------------------------------------------
>A.linkianum_BAMTp      TTATAAGTACTTCAATTAAATAACTTTTTTTGGGATCAAAATATGTACAC

>A.majus_BAMTp          --------------------------------------------------
>A.linkianum_BAMTp      AAACTCACATAGTGTAGGAGAGTCTTTATGTAAATCATCATCTGCAATTA

>A.majus_BAMTp          --------------------------------------------------
>A.linkianum_BAMTp      TTTTACTTGTTAGTTGACCAATTATTAAAGAAAAAACTAATCATTTAGTT

>A.majus_BAMTp          --------------------------------------------------
>A.linkianum_BAMTp      AACATTGACGTTATGTTAGCATTTAATTTAGTTTATTGACTTATATGAAC

>A.majus_BAMTp          --------------------------------------------------
>A.linkianum_BAMTp      ACTTTTTTCTTGATTTTTTTGTATTACTTGCTTTTTCAAAAAACTATTAG
```

```
>A.majus_BAMTp          --------------------------------------------------
>A.linkianum_BAMTp      TTAACAGGACATAGATTTTTTATAACCAATTTATACTACTTTATCTAATG

>A.majus_BAMTp          --------------------------------------------------
>A.linkianum_BAMTp      TCAGATAACTTATATAGATCCTTAATTTCTTATGTATGATTAAATGTCCT

>A.majus_BAMTp          --------------------------------------------------
>A.linkianum_BAMTp      GAAAATAATTTTATATTTATTTATTAAATATTTGTTTACTTGTGCATTTA

>A.majus_BAMTp          --------------------------------------------------
>A.linkianum_BAMTp      AAATAGTTATCCTTGATGTCAATTTACTTTAGCCCTCCCCAAATTAAATT

>A.majus_BAMTp          ------------------------TAACTCGAATTGAGCAATCATACTCG
>A.linkianum_BAMTp      TCTGAATACGCCCCTGTTTGCATCTAACTCGACTTGAGCAATCATACTCG
                                        *** ** ********* *****************

>A.majus_BAMTp          TGATAGATAAGTTAATTTCACATGTATTACTCTTTTTAAATTGACCAAAA
>A.linkianum_BAMTp      TGATAGACAAGTTAATTTCACATGTATCACTGTTTTCAAATTGACCCAAA
                        ******* ******************* *** **** ********* ***

>A.majus_BAMTp          AAAAATGTCATACTATAAGATTAAACTGGGAAGTGGGGGGCGTATCTACT
>A.linkianum_BAMTp      AAAAAAGTCATACTATAAGATTAAA-TGGGAAGTGGGGGGCGTATCTACT
                        ***** ******************* ************************

>A.majus_BAMTp          TAGGGTACTCCAGCCCTCTTATATATATAATGTATTATATATATATATAT
>A.linkianum_BAMTp      TAGGGTCCAGCTAGCCTCTTATATATATA---TAT-ATATATATATATAT
                        ****** *  *   ***************   *** **************

>A.majus_BAMTp          ATACACAAAAAGGGAAAAAAATATTTATTGAAATTGTTAAAGGTTACAAA
>A.linkianum_BAMTp      ATATTCAAAAAGGAAAAAAAATATTTATTGAAATTGTTAAAGGTTACAAA
                        ***  ******** ************************************

>A.majus_BAMTp          TTCAATTTTCAATAAAGCTTATCGACAAAATCCCAAACTATAGAAAATTA
>A.linkianum_BAMTp      TTCAATTTTGAATAAAGCTTATCGACAAAATCCCAAACTATAGAAAATTA
                        ********* ****************************************

>A.majus_BAMTp          AGTTCTCTTCAACGCCCAACAATCAAACGTATTGACAAAATCTTTATGTT
>A.linkianum_BAMTp      AGTTTTCTTCAACGCCCAACAATCAAATGTATTGACAAAATCTTTATGTT
                        **** ********************** **********************

>A.majus_BAMTp          CTTGGTAAAAATTTGTAGTAGTCTGAAATTTTAATAATGTGACAAAGGAA
>A.linkianum_BAMTp      ATTG-TAAAATTTTGGAGTAGTCTGAAATTTTAATAATGTGACAAAGGAA
                         *** ***** **** **********************************

>A.majus_BAMTp          CATAAGTAAGCAAATAGTGAA----AAACTAAATATATATTAACTCATAT
>A.linkianum_BAMTp      CATAAGTAAGCAAATAGTGAATAGAAAACTTAATATATATTAACTCATAT
                        *********************    ***** *******************

>A.majus_BAMTp          TTTCAATTATTATATTCATGTGTTGTTAAGTGATTAATATATCATTTACC
>A.linkianum_BAMTp      TTTCAAATATTATATTCATGTGTTGTTAAGTGATTAATATATCATTGACC
                        ****** ************************************** ***

>A.majus_BAMTp          ATTAGTATTTTTGACTTTTTCAATTTTTTGTTATTTAATTTTGTTTTTGT
>A.linkianum_BAMTp      ATTAGTATTTTTAACCTTCAATTTTTTTTGTTATTTAATTTTGTTTTTGT
                        ************ ** **     ***************************
```

```
>A.majus_BAMTp          GTTATATATCACTTTTATATTATTAT-TATTACTTGTTTTACCAAAAAAA
>A.linkianum_BAMTp      GTTATATATCACTTTTATATTATTATGTATAACTTGTTTTACCAAAAAAA
                        ************************** *** *******************

>A.majus_BAMTp          TATTTA-AATTTCTTATATTGTGTTTCTTATATTTTAATTTCTTAAATTA
>A.linkianum_BAMTp      AATTTTTAAATTCTTATATTGTGTTTCTTATATTTTAATTTCTTAAATTA
                         ****   ** ****************************************

>A.majus_BAMTp          CATACCTTTTTCATAGTACTTAAAAAATATATCCGTTGGATTAGTCCAAC
>A.linkianum_BAMTp      CATACTTTTTTCATAGTACTTAAAAAATATATCCGTTGGATTAGCCCAAC
                        ***** ************************************** *****

>A.majus_BAMTp          GCAGACTGAATTCCTGGATACGCCCTCGTGTACATACGTGCTATATTAGA
>A.linkianum_BAMTp      GCAGACTGAATTCCTGGATACGCCCTCGTGTACATACGTGCCATATTAGA
                        ***************************************** ********

>A.majus_BAMTp          AAATAGGAAGAGCAAACTATCAAGTATTTGTAAGTTGTAGCTGCCGAACA
>A.linkianum_BAMTp      AAATAGGAAGAGGAAACTATCAAGTATTTGTAACTTGTAGCTGCCGAACA
                        ************ ******************** ****************

>A.majus_BAMTp          ATTTGGTTACTGAATTATATATATATATGTGAATCTATTTGCTGGTAAGC
>A.linkianum_BAMTp      ATTTGGTTATTGAATTATATATA----TGTGAATCTATTTGTAGGTAAGC
                        ********* *************    **************  *******

>A.majus_BAMTp          GTTAGAAGTTATACCCGAGTAGAAGAATTAA
>A.linkianum_BAMTp      GGTAGAAGTTATAACCGAGTAGAAGAATTAA
                        * *********** *****************
```

Supplementary Figure 2. *A. majus* vs. *A. linkianum BAMT* promoter. Key color: green-insertions, blue-deletions, yellow-repetitions.
